# eCAT: Online electronic lab notebook for scientific research

**DOI:** 10.1186/1759-4499-1-4

**Published:** 2009-10-29

**Authors:** Nigel H Goddard, Rory Macneil, Jonathan Ritchie

**Affiliations:** 1 School of Informatics, University of Edinburgh, 10 Crichton Street, Edinburgh, EH8 9AB, UK; 2 Axiope Limited, 24 Fountainhall Road, Edinburgh EH9 2LW, UK; 3 Axiope Limited,24 Fountainhall Road, Edinburgh EH9 2LW, UK

## Abstract

**Background:**

eCAT is an electronic lab notebook (ELN) developed by Axiope Limited. It is the first online ELN, the first ELN to be developed in close collaboration with lab scientists, and the first ELN to be targeted at researchers in non-commercial institutions. eCAT was developed in response to feedback from users of a predecessor product. By late 2006 the basic concept had been clarified: a highly scalable web-based collaboration tool that possessed the basic capabilities of commercial ELNs, i.e. a permissions system, controlled sharing, an audit trail, electronic signature and search, and a front end that looked like the electronic counterpart to a paper notebook.

**Results:**

During the development of the beta version feedback was incorporated from many groups including the FDA's Center for Biologics Evaluation & Research, Uppsala University, Children's Hospital Boston, Alex Swarbrick's lab at the Garvan Institute in Sydney and Martin Spitaler at Imperial College. More than 100 individuals and groups worldwide then participated in the beta testing between September 2008 and June 2009. The generally positive response is reflected in the following quote about how one lab is making use of eCAT: "Everyone uses it as an electronic notebook, so they can compile the diverse collections of data that we generate as biologists, such as images and spreadsheets. We use to it to take minutes of meetings. We also use it to manage our common stocks of antibodies, plasmids and so on. Finally, perhaps the most important feature for us is the ability to link records, reagents and experiments."

**Conclusion:**

By developing eCAT in close collaboration with lab scientists, Axiope has come up with a practical and easy-to-use product that meets the need of scientists to manage, store and share data online. eCAT is already being perceived as a product that labs can continue to use as their data management and sharing grows in scale and complexity.

## Background

### eCAT

This article describes the background to and evolution of eCAT, an electronic lab notebook (ELN) developed by Axiope Limited. eCAT is the first online ELN, the first ELN to be developed in close collaboration with lab scientists, and the first ELN to be targeted at researchers in non-commercial institutions.

### Electronic laboratory notebooks

An electronic lab notebook (also known as electronic laboratory notebook, or ELN) is a software program designed to replace paper laboratory notebooks [1]. Compared with paper lab notebooks, ELNs bring many benefits, including an improved search capability, support for collaboration amongst many users, and enhanced security [1]. ELNs have been adopted widely in the pharmaceutical industry over the past 15 - 20 years, where penetration among researchers is now over 50%. With features like audit trails and electronic signatures, ELNs now play a vital role in IP management and protection, and compliance with regulatory requirements such as 21 CFR part 11.

Over the past five years a number of trends have been evident in the evolution and usage of ELNs in industry, including:

1. The emergence of specialist ELNs to handle areas like quality control and quality assurance

2. The spread of ELNs beyond the pharmaceutical industry into other research intensive 'process industries' like food and beverage, agriculture and petrochemicals

3. The convergence of ELNs with their sister product, laboratory information management systems (LIMS [2])

4. Demand for highly specialized ELNs that have been tailored for particular kinds of research

### Need for online notebooks

Notwithstanding the proven utility of ELNs in the scientific research process, *only 4% of researchers in non-profit institutions use ELNs *[3]. The primary reason is cost: ELNs sold to industry are priced at $1,000s per seat, and often come with bespoke development and add ons costing $100,000s on top of the per seat fee. This puts them out of reach of the vast majority of non-profit institutions and their researchers. Inertia and attachment to the familiarity of the paper lab notebook probably also help account for the low penetration of ELNs in non-profit institutions.

Additional impediments to adoption of the ELNs sold to Pharma in non-profit research settings are their lack of accessibility and flexibility. These ELNs are architected on a client-server model. This, combined with the specialization that results from their normally being tailored for specific research purposes, makes them complex and inflexible. Scientists using them are dependent on IT for support and are unable to input their own design ideas into the research process. This is ill suited to the distributed research model prevalent in non-profit settings, where the relatively autonomous individual lab is the dominant model. Collaboration among labs within and between institutions is common, but these larger groupings maintain a bottom up ethos rather than the departmental or corporate structure prevailing in industry.

Scientists working in non-profit institutions need user friendly and flexible tools that can be learned and used with minimal IT support. Web-based applications make it possible to bring to scientists working in non-profit settings the benefits ELNs have brought to corporate research. These include improved search capability, support for collaboration amongst many users, enhanced security, an audit trail, electronic signatures, and a permission system which permits controlled sharing of data within and between labs.

There are two reasons for this. The first reason is technical. Web applications are accessible 24/7 to anyone with access to a web browser, providing a platform for sharing data and information among individuals and groups who are located anywhere. The second is commercial. Web-based applications often can be delivered and supported at a fraction of the cost of IT-intensive client-server applications. So, with web applications now firmly established as a product category, the framework is in place to offer scientists in non-profit settings an application with the basic functionality of commercial ELNs at a fraction of the cost.

### eCAT

Axiope saw the opportunity to deliver a functional but affordable web based ELN to scientists back in 2006. Realization of the need for a web-based ELN arose from combining feedback from users and prospective users about the limitations of a predecessor product, described below, and observing other kinds of web-based applications that were beginning to appear.

eCAT's predecessor, Catalyzer, enjoyed limited success when it was in the market from 2003 to 2006. Catalyzer was an XML-based user configurable database which had been tailored for managing scientific inventory and integrated with a barcoding application. The main criticisms received from users were:

1. lack of scalability: Catalyzer became unacceptably slow after 10,000 records were added to a catalog

2. poor interface: Catalyzer lacked an intuitive, familiar front end

3. client-server model: Catalyzer was not web-based

4. at $1,000 per seat Catalyzer was too expensive for most non-profit settings

As these criticisms were giving rise to the realization that a fundamental remake of the product would be necessary, early web-based collaboration applications were beginning to be adopted, including by some scientists. Of particular relevance was the rise of the wiki. Axiope began following and learning about a number of wiki offerings, and eventually determined that a great deal could be learned from them in terms of both the kind of collaboration wikis permitted and their business model. Ultimately Axiope came to believe that Atlassian's Confluence, the leading 'enterprise', i.e. institutional scale, wiki, was most relevant to our plans to introduce a web-based ELN. Confluence's users included some NIH institutes and a wide range of universities, and its pricing model -- roughly $100 per user per year - seemed appropriate for the non-profit scientific users Axiope was targeting.

Between late 2006 and the autumn of 2007 the basic shape of what was to become eCAT began to crystallize: a highly scalable web-based collaboration tool that possessed the basic capabilities of commercial ELNs, i.e. a permissions system, controlled sharing, an audit trail, electronic signature and search, and a front end that looked like the electronic counterpart to a paper notebook. Consideration was given to integrating eCAT with a wiki, but it did not seem sensible to limit users to a single wiki offering and in the end a general purpose HTML rich text editor was built into eCAT instead. TinyMCE was chosen because of its industry standard approach and rich feature set which allows eCAT users to create complex and detailed experimental write-ups inside their web browser.

From the moment the idea for eCAT began to emerge, and throughout its design and beta testing, Axiope remained focused on the core principle on which eCAT's predecessor Catalyzer was based: 'by scientists for scientists'. To ensure that eCAT was a product scientists would adopt widely it was crucial that they be closely involved in its development. To that end a key milestone was reached in October 2007, when a group at the FDA's Center for Biologics Evaluation & Research, which was looking to adopt an ELN, came across Axiope. The FDA group liked a mock up of eCAT and the explanation of plans for its development, and over the next year provided ongoing feedback as development of the code gathered pace (see below for details).

The beta version of eCAT released in September 2008 had been developed to the spec provided by the FDA group. The group was particularly helpful in providing feedback from scientists on the design and look and feel of eCAT's front end. Useful feedback was also incorporated from groups at Uppsala University and Children's Hospital Boston, as well as Catalyzer users like Alex Swarbrick's lab at the Garvan Institute in Sydney, Australia and Martin Spitaler at Imperial College, London.

## Implementation

### Features/advantages of eCAT

#### Features

1. Runs entirely in the web browser - no client software needs to be installed.

2. The server runs on industry standard tools and works across all three major operating systems - Windows, Linux and OSX.

3. Powerful data entry and editing capabilities.

4. Ability to work with existing file storage solutions or to permit the upload of files to new storage areas.

5. Simple yet powerful search with the ability to customize what you are searching for in great detail

6. Export facilities to the popular formats of CSV, Excel and XML.

7. Powerful permissions system to control who has access to what data. This extends to several other permissions such as edit and download control.

8. A complete set of user and group functionality to control access by group, individual or both.

9. "Fully typed data" with the common types (String, Text, Radio, Date, Time etc) fully supported.

10. Class editing capabilities allow users to create their own classes (collections of data types) to manage their data in their own way.

11. Ability to sign and authorize projects and experiments for regulatory and compliance purposes.

12. Familiar interface - a tree like in Windows to browse the data and an editing interface similar to a traditional paper lab notebook.

13. Support for the import and thumbnailing of many of the more common scientific image formats, such as LSM, TIFF and STK.

14. Administrative and audit features.

#### Advantages

**Figure 1:**
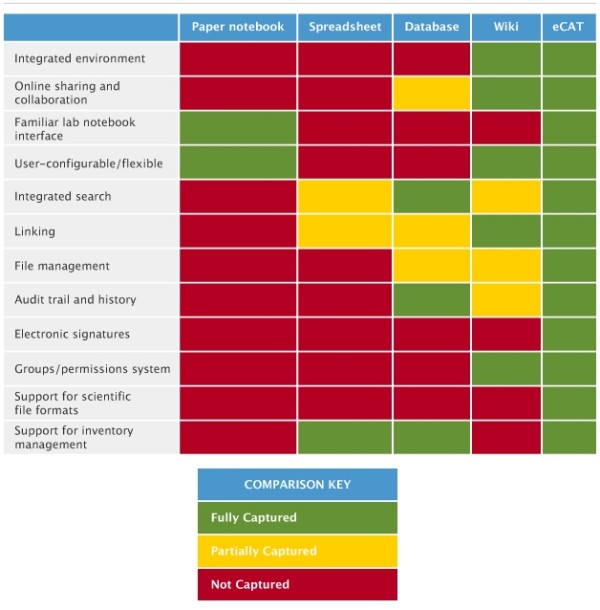
**eCAT compared to alternatives**.

eCAT's advantages, compared with other alternatives scientists might considering using, are set out in Figure 1.

#### Design of the software

The initial design of eCAT focused on its kernel and attempted to address the scalability problems of the previous Catalyzer kernel while retaining the ability to restore the data in an XML format. Once that was complete a variety of backing storage implementations were considered before the database and XML backend combination now in use was selected. This was chosen because of the need to have a capability for users to define what would be "database tables" in a more traditional database based web application.

Once the kernel that allowed for the necessary scalability and flexibility had been established much of the actual web applications design was derived from the use of the Spring and Hibernate frameworks.

As noted above, the user interface was designed after extensive communication with scientists and is in continual evolution even after the first release with the regular addition of new and the improvement of existing features.

#### Implementation technologies

eCAT is written entirely in Java and uses industry standard tools for deployment - Tomcat or Apache and MYSQL or a similar database. A web application framework called Appfuse was used initially to bring together some of the standard java technologies such as Spring and Hibernate. Appfuse, Spring and Hibernate are all open source programming technologies. Spring lets you work quickly and easily to build a web-based application, Hibernate lets you talk to databases and Appfuse pulls together a number of technologies, including Spring and Hibernate, into a cohesive whole.

## Results and Discussion

### Beta release overview

More than 100 individuals, labs and larger groups - consortia, departments, centers, etc. - participated in the beta testing of eCAT between September 2008 and June 2009. Testers came from a wide geographical spread, including North America, Europe, the Middle East, and Asia. During this period a total of four different versions were released.

The following were the most important changes that were made in the releases:

The first release included an initial attempt at the lab notebook interface. As noted above, much of the design was based on consultation with real scientists, in particular the design of the Project and Experiment templates and the signing and authorizing features.

The second release refined the interface and added a set of new features. The upload process was significantly simplified, and more power was given to individual users with the ability to edit their own preferences and, more importantly, the addition of support for class editing for users - this had previously been an admin level function.

In the third release performance was improved, and the application was scaled up to support millions of records. All parts of the system were improved, with the tree performance being the most obvious user level improvement.

The fourth release was a final bug fix in preparation for the first commercial release.

### Reactions to eCAT

Of those who actively tested eCAT, the majority reported positive feedback. This is described in more detail below. Three reasons were given by the small number of testers who said they would not be inclined to adopt eCAT in its beta version:

1. A preference for a more free form environment where information is structured as a result of tagging.

2. A desire to be able to make all data in eCAT public.

3. Lack of support for LDAP integration.

The majority of the dozens of testers who commented on eCAT were positive. Many of their suggestions were incorporated into eCAT during the upgrades that were made during the beta period, and most of the rest will be included in the improvements planned for releases in the first six months after the commercial release. These are described below.

### Observations about adoption and usage from the beta testing

A revamped Axiope website was launched to coincide with the beta testing of eCAT. The website included an online video structured as a step by step guide to using eCAT, and a detailed but practical user guide in the Help section to make it possible for most users to get started with eCAT making use of these materials, without the need for direct training. The videos proved particularly popular; a high percentage of people visiting the site watched the videos, and a number of testers reported that the videos had been useful in introducing eCAT.

eCAT was offered in two forms. Individual testers were set up with accounts on a *service hosted by Axiope*. Most groups were set up with a version of eCAT installed on a lab or institutional server. For these *'customer managed' installations *an inhouse administrator then set up accounts for individual users. Where requested, for example by the FDA and Children's Hospital Boston, a demo was given to a core group of initial users. In other cases, the adoption process was left to the testing lab or institution. Some customer managed users adopted a very structured approach to encouraging adoption, whereas others took a more freeform approach.

A good example of the structured approach is the modENCODE consortium. The National Human Genome Research Institute has designated the modENCODE (model organism ENCyclopedia of DNA Elements) Project to try to identify all of the sequence-based functional elements in the *Caenorhabditis elegans *and *Drosophila melanogaster *genomes. The group which has adopted eCAT focuses on *C. elegans *and is a collaboration between labs at University of North Carolina, University of Cambridge, University of California, Berkeley, University of California, San Diego, NimbleGen, the Dana Farber Cancer Institute, and the Weizmann Institute.

ModENCODE designated two 'champions', Huang Pham of Lawrence Berkeley Labs and Morten Jensen of the University of North Carolina Chapel Hill. After viewing a demo of eCAT Hoang and Morten did a considerable amount of their own testing, including asking questions and making comments. They developed their own instructional materials including a powerpoint presentation and a video, and used these in introducing a few other users in the group to eCAT. They then rolled eCAT out to a larger group of users. They kept the larger group informed of developments throughout the process and held several online meetings and briefings at key points. These were interactive, and included a chance for users to provide feedback about likes and dislikes. During the process they also communicated regularly with Axiope, getting help with problems and questions that arose, making suggestions for feature additions and changes, and collating feedback from Axiope about which of these requests were likely to be implemented and on what time scale.

### eCAT launch

The commercial version of eCAT was launched in July 2009. The launch version includes the upgrades and improvements described above. In addition, the Axiope website has been completely revamped. Two aspects of the revamp are worth emphasizing.

First, the basic mechanics of delivering eCAT commercially are provided for. eCAT is being offered in three versions:

1. A Team Hosted version

2. An instance of eCAT installed on the customer's server

3. A Personal version (free of charge) on a server hosted by Axiope

A trial version is available free for a month, and an e-commerce facility has been added to enable purchase by credit card.

Second, reflecting what was learned during the beta, a significant redesign of the site has been implemented. This is aimed at making it even easier for prospective users to understand eCAT and to learn how to use eCAT, and for users to get the most out of eCAT. This has been done, first, through making the following additions to the website:

1. Two new videos overviewing the main activities that can be undertaken with eCAT:

1.1 Editing experiments

1.2 Managing inventory

2. A new section of the site called 'Learn', which includes subsections explaining

2.1 What can you do with eCAT

2.2 Who eCAT is for

2.3 eCATs features

2.4 Supported plugins and image formats

2.5 eCAT compared to alternatives like paper, spreadsheets, databases & wikis

2.6 User - feedback Q&As

*In addition*, there is now a link from the home page to a separate site; *eCAT community*. *eCAT community *is intended to take the level of user feedback and input into eCAT's ongoing development to a new level. *eCAT community *hosts the Axiope blog, and several forums. It also provides a messaging function to enable conversations between eCAT users at different institutions. And there is a space for upload of instructional and explanatory materials, like the modENCODE powerpoint noted above, that users themselves have created. It isAxiope's strong belief that the best person to teach a scientist how to use and get the most of our eCAT in their research is another scientist who is using eCAT. *eCAT community *is intended as a platform to let that kind of communication and exchange of information flourish.

### Potential extensions and improvements

Extensions and improvements are planned for eCAT in a variety of areas.

#### Features

Over the first six months after the commercial release in June, a number of feature improvements are planned. These include:

• Better handling of tables, including editing in table views, importing spreadsheets and the ability to edit multiple records at the same time

• Improvements to the search form, for example by adding multiple classes for search selection

• Image annotation

• Automatic saving and loading of data straight from other applications, e.g. Word and powerpoint

• Permitting specification of which classes are displayed as menu entries

• Removing restrictions on record naming

#### Delivery

We plan to incorporate LDAP, to facilitate institutional users integrating eCAT into their existing IT environment.

#### Extensibility/interaction with other applications

After an initial breathing space of around six months to ensure that eCAT is bedded down, enhancing the ability of eCAT users to interact with other applications will be a high priority. Creating an API is top of the list, which also includes improved import/export capabilities, possibly integration with wikis, and smoother interface with other applications that scientists use in their research.

#### Mobility

Also planned for 2010 is a major initiative relating to mobility, which will enable data entry and search in eCAT through smartphones.

#### Website

The plan is for *Axiope Community *to evolve into an important source of information and support for users, and improvements and changes will be made on a regular basis.

#### By scientists, for scientists

The close involvement of users which has characterized eCAT's initial inspiration, and subsequent design and development to date, will continue to inform every aspect of e-CAT's development going forward.

## Competing interests

The authors are all shareholders in Axiope, and as such would benefit indirectly from positive responses to this article. Over the past five years each of the authors has received fees and/or a salary from Axiope.

## Authors' contributions

The authors contributed equally to this work.
